# Hemodynamic assessment by neonatologist using echocardiography: Primary provider versus consultation model

**DOI:** 10.1038/s41390-024-03248-7

**Published:** 2024-05-22

**Authors:** Shahab Noori, Rangasamy Ramanathan, Satyan Lakshminrusimha, Yogen Singh

**Affiliations:** 1 Fetal and Neonatal Institute, Division of Neonatology, Children’s Hospital Los Angeles, Department of Pediatrics, Keck School of Medicine, University of Southern California, Los Angeles, CA USA; 2 Division of Neonatology, Department of Pediatrics, LAC+USC Medical Center, Keck School of Medicine of USC, Los Angeles, CA USA; 3 Department of Pediatrics, UC Davis Children’s Hospital, UC Davis Health, Sacramento, CA USA; 4 Department of Pediatrics - Division of Neonatology, Loma Linda University Children’s Hospital and Loma Linda University School of Medicine, Loma Linda, CA USA

## Abstract

Hemodynamic instability is very common in sick neonates and the currently used traditional hemodynamic monitoring tools lack sensitivity and specificity. Hemodynamic evaluation on echocardiography can provide direct information regarding the pathophysiology causing the hemodynamic instability and help the bedside clinician in making a personalized treatment approach based upon the deranged pathophysiology. Assessment of cardiac function and hemodynamics is essential in the management of neonates with cardiorespiratory failure, and hence neonatologist-performed echocardiography is becoming an essential tool in modern neonatal care. Depending on the level and size of the NICU, there is a daily need for echocardiography, and for a subset of sick infants, serial echocardiographic assessments are warranted. Comprehensive guidelines for neonatologists performing echocardiography and targeted neonatal echocardiography have been published providing a framework for training and quality assurance. There has been a significant interest among the providers to learn echocardiography skills. This manuscript explores the various needs of neonatal care providers around echocardiography, the current challenges neonatologists face in learning echocardiography, and how they, especially neonatal fellows, can learn these important skills during their training.

## Introduction

Clinical evaluation and traditionally used laboratory markers are the indirect proxy-parameters of cardiovascular well-being. The limitations of both clinical and laboratory assessments regarding the adequacy of circulatory function in neonates, especially during the early postnatal transitional period, have long been recognized.^1,2^ Echocardiography allows for a direct assessment of cardiovascular function and significantly enhances the clinician’s ability to diagnose and manage cardiovascular insufficiency in the neonatal intensive care unit (NICU).

## History and evolution

Functional echocardiography was introduced in NICUs in the 1990s.^3^ As the name indicates, the focus was the assessment of the function rather than the structure of the heart, and the choice of the name was in part to differentiate it from cardiologist-performed echocardiography to rule out congenital heart defects (CHD). At the time, the main focus of functional echocardiography was the assessment of the adequacy of systemic blood flow during the transitional circulation among very preterm infants, a population at high risk for brain injury secondary to cerebral ischemia or intraventricular hemorrhage (IVH). With the increased interest in a more enhanced assessment of hemodynamics among neonatologists in the 2000s, the scope of functional echocardiography started to grow. Along with expanding its application in the NICU, different terminologies such as Point-of-Care (POC) echocardiography and Targeted Neonatal Echocardiography (TNE) were introduced as a substitute for functional echocardiography to emphasize various aspects of the procedure.^4^ Neonatologists Performed Echocardiography (NPE) in Europe, focuses on the provider performing the procedure to differentiate it from cardiologist-performing studies and emphasizes the limited structural assessment of the heart as one of the goals is to differentiate between normal and abnormal heart.^5–7^ Other terms, such as Clinician-Performed Ultrasound (CPU) used in Australia demonstrate the limited assessment of the heart on ultrasonography. While the scope of echocardiography performed by the neonatal care provider varies in different countries and even in different NICUs within a country, in this article, we use NPE and TNE interchangeably to refer to the echocardiography performed and interpreted by the neonatologist.

## Various clinical needs

Although assessment of the hemodynamics beyond physical exam can aid in the diagnosis and management of cardiovascular compromise in neonates, there are different clinical scenarios in which the extent of skills needed to perform echocardiography and interpret results vary. For example, ruling out significant pericardial effusion requires a more basic echocardiographic skill than the management of pulmonary hypertension in a neonate with the vein of Galen malformation, where a more advanced skill and knowledge of hemodynamics are needed.

## Hemodynamic evaluation using TNE

**Fig. 1:**
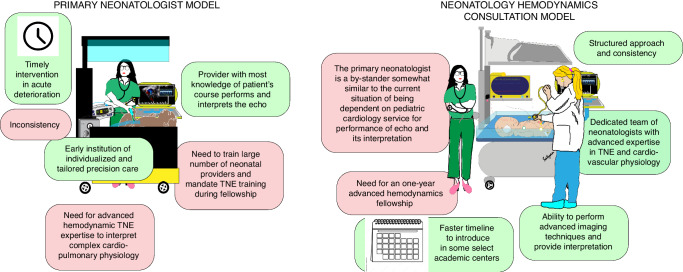
Hemodynamic assessment models in the NICU. The pros and cons of the primary neonatologist model (left panel), where hemodynamic evaluation is performed by the primary neonatal care provider, versus neonatology hemodynamics consultation model (right panel), where hemodynamic evaluation is performed by the specialist neonatal hemodynamic team are shown.

Assessment of cardiac function and hemodynamics is essential in the management of neonates with cardiorespiratory failure. Depending on the level and size of the NICU, there is a daily need for echocardiography, and for a subset of sick infants, serial echocardiographic assessments are warranted. Although some of these needs, including ruling out congenital cardiac diseases (CHD), are met by cardiology service, there are clinical situations where the neonatologist caring for the patient at the bedside is better positioned to perform these assessments. These clinical situations include acute decompensation when immediate assessment is needed and cases when serial evaluation is necessary to titrate fluid therapy, vasoactive medications, inotropes, pulmonary vasodilators, and ductal constrictors/dilators. Table 1 describes the common clinical indications for hemodynamic assessment using TNE. There are two models of hemodynamic evaluation using TNE: primary neonatologist and neonatology hemodynamic consultation team (Fig. 1).

**Table 1:** The common clinical indications for hemodynamic assessment using neonatologist-performed echocardiography.

Shock
Hypotension
Metabolic acidosis
Narrow pulse pressure
Low urine output
Escalation of cardiovascular medications
Cardiac arrest
Hypertension
Hypoxemic respiratory failure
Extremely preterm infant during transition
Clinical signs of patent ductus arteriosus (PDA)
Post-PDA ligation or transcatheter closure
Conditions associated with cardiovascular insufficiency (e.g. congenital diaphragmatic hernia)

### Primary neonatologist model

In this model, the neonatologist, who is the primary physician, performs and interprets the TNE. This is the traditional and most widely practiced model. There are several advantages to this model. The primary neonatologist is the physician most familiar with the patient’s history and clinical presentation, progression of the disease, the impact of various treatments, and precise questions needing to be answered by using TNE. Therefore, the primary neonatologist is better positioned to incorporate the TNE findings with all other pieces of information to shed light on the underlying pathophysiology and formulate a treatment strategy. In other words, the primary neonatologist is best suited to provide individualized and tailored precision care. Another advantage is that the hemodynamic assessment can be performed at any time without delay. The examination can be focused and relatively brief so that evaluation and management can be instituted in a time-sensitive setting. This is especially important in the presence of acute clinical deterioration. However, there are disadvantages to this model as compared to a neonatology hemodynamic consultation team. Although the number of neonatologists with TNE has significantly increased over the past decade, they still represent a small percentage of academic neonatologists. Training all currently active neonatal providers and offering TNE training during neonatal-perinatal medicine fellowship can be challenging in countries such as the USA. Furthermore, a small subset of patients with cardiorespiratory failure has a complex pathophysiology that requires advanced hemodynamic expertise to interpret the TNE findings correctly. Lastly, there can be inconsistency of the service delivered because of varied TNE skills among providers and their availability.

### Neonatology hemodynamic consultation model

In this model, a neonatology hemodynamic consultation team performs and interprets the TNE and makes recommendations to the primary neonatologist. This is a newer model and is only available in a small number of academic centers with a significant hemodynamic focus. The biggest advantage of this model includes having a dedicated team of neonatologists who have advanced expertise in echocardiography and cardiovascular physiology to evaluate the patient and perform and interpret TNE. They can expand the TNE assessment to include more advanced measurements such as tissue Doppler imaging and strain rate. Another advantage of this model lies in having a small group responsible for performing and interpreting TNE. This allows for a more structured approach to TNE and accumulates real-life TNE experience in a shorter time. This model improves the consistency of the TNE and hemodynamic evaluation services. In addition, this model and the associated infrastructure can support the one-year hemodynamic-focused fellowship and would also be ideal for training the newer generation of neonatologists in developing expertise in hemodynamics. Among the disadvantages of this approach is replacing the primary neonatologist with a physician who has not been caring for the patient. This is important as the whole idea of performing TNE by the neonatologist rather than by the cardiology service is that the treating physician is in a better position to perform and interpret the functional echocardiography. The other limitation of this model is that a core group of neonatologists with such expertise is needed to provide day and night coverage, something that is currently only possible in a handful of academic centers. Currently, it is not possible to have such a model in most of the academic NICUs, and this essentially excludes non-academic NICUs. As the vast majority of preterm and sick term infants are cared for by neonatologists in non-academic NICUs, the benefits of TNE will only be realized for a tiny fraction of the population. Therefore, while the consultation model is likely more effective in unraveling the underlying pathophysiology in the most complex patients, due to its limitations, it has significantly less direct impact on the neonatal population as a whole until this model becomes widely available across most of the academic and non-academic NICUs.

Considering the advantages and disadvantages of each model, it is clear that the two models are complementary, and both are needed for TNE to fulfill its primary goal of improving the outcomes in sick neonates in the NICU.

## Cardiac POCUS

Compared to TNE, cardiac point-of-care ultrasound (POCUS) is less well-defined.^8^ It generally refers to ultrasonography performed by the primary caregiver (e.g., a neonatologist) to answer a specific clinical question and usually in an emergency situation, for example, ruling out poor contractility or pericardial effusion as the cause of the cardiovascular collapse (Video 1). Compared to TNE, less amount of training and skills are needed to perform and interpret cardiac POCUS. However, depending on the definition of cardiac POCUS, there can be a significant overlap between TNE and cardiac POCUS. For example, in the International guidelines on POCUS for critically ill neonates and children issued by the POCUS Working Group of the European Society of Paediatric and Neonatal Intensive Care, cardiac POCUS has a broader scope than what was described above.^9,10^ It includes qualitative and semi-quantitative assessment of preload, fluid-responsiveness, and contractility, evaluating for patency of the ductus arteriosus, and recognition and limited assessment of pulmonary hypertension. However, this international guideline was developed for use in both neonatal and pediatric intensive care units across the countries and centers with a very significant limitation in the availability of pediatric cardiology and TNE services. In countries like the USA, where most centers have access to pediatric cardiology or TNE services in a reasonable time, neonatal cardiac POCUS should be used only for limited indications such as assessment of cardiac contractility, detection of pericardial effusion or cardiac tamponade, and rapid evaluation of shock to determine the need for fluid versus inotropic therapy (Table 2). Although except in the severe case, qualitative and semi-qualitative interpretation of echocardiographic indicators of preload and contractility is less reliable than a quantitative evaluation using comprehensive TNE, nevertheless, they provide a direct evaluation of the cardiac function and greatly supplement the clinical examination, especially in emergency situations.

**Table 2:** Indications for the cardiac point-of-care ultrasound in neonates.

Rapid assessment of shock
Qualitative assessment of cardiac contractility
Rapid assessment of cardiac tamponade and pericardial effusion
Guide pericardiocentesis for cardiac tamponade
Verification of central catheter placement

Recently, guidelines for using POCUS in emergencies in the NICU referred to as “Crashing Neonate Protocol” and SAFE-R protocols for acutely decompensating infants have been proposed.^11,12^ In these protocols, in addition to a limited ultrasound of the head, abdomen, and chest, cardiac POCUS is performed to identify some of the major causes of acute cardiorespiratory failure. The cardiac POCUS components are recognition of pericardial effusion and tamponade, poor contractility, underfilled heart, and pulmonary hypertension.

As the training and scope of neonatal cardiac POCUS are very different from TNE or point-of-care echocardiography, in this paper we will focus on echocardiography training only.

## Neonatologist performed echocardiography – TNE Training

Although there is a consensus that echocardiography performed by neonatologists (TNE/NPE) and cardiac POCUS are both valuable in the management of neonates with cardiorespiratory failure, there is no widely accepted structure or process to acquire the skill and, more importantly, to verify competency.^13^ The expert opinion and consensus statements in the last decade have provided some framework and guidelines on training and quality assurance.

**Fig. 2:**
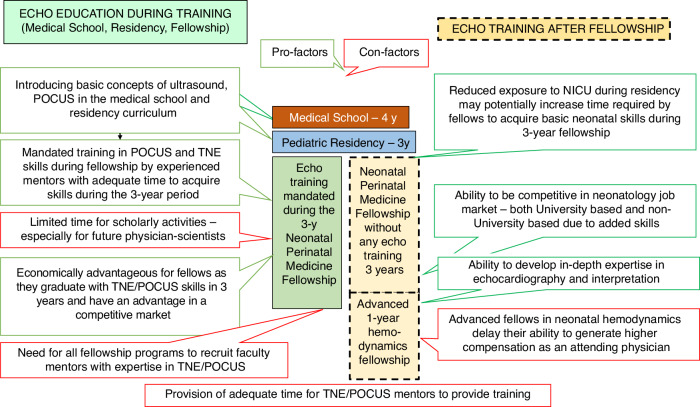
Echocardiography training. The pros and cons of learning neonatologist performed echocardiography during and after neonatal fellowship training are shown.

Some of the challenges in standardizing echocardiography training stem from the regional and institutional differences in the availability of resources and expertise to support a TNE or cardiac POCUS program. Although there are many courses and workshops on the subject providing the initial steps in learning echocardiography, one needs to spend at least 6–12 months as a mentored trainee to acquire the necessary TNE skills in obtaining images and interpreting the findings. This may be achieved during or after the neonatology fellowship (Fig. 2), the scope of which warrants further exploration as there is an increased demand to develop such skills and limited availability of the resources (trainees’ and mentors’ time and availability of the infrastructure).

### Training during fellowship

Training during the fellowship has several advantages. Fellows have three years to learn and to be evaluated. If TNE becomes a part of fellowship training, the impact on the expansion of echocardiography knowledge and skills in the neonatology workforce and, by extension, on neonatal patient care will be highly significant. Ideally, basic principles and use of ultrasound should be taught in medical school and be included in the pediatric residency curriculum so that incoming fellows have some basic knowledge of POCUS/TNE. However, there are several limitations. Although increasing, the number of fellowship programs with the infrastructure and expertise to support a fellowship TNE program is still low. Even in the programs with TNE expertise, the demand for substantial time commitment from the TNE faculty poses a significant barrier, especially in smaller programs where only one or two TNE experts are on staff. The other challenge is completing neonatology learning objectives for the fellow in a timely manner.

Here, we briefly describe some of our experiences training neonatology fellows in our programs. At Los Angeles County Hospital, the University of Southern California, and Children’s Hospital Los Angeles, opportunities for learning echocardiography for neonatology fellows have existed since 2000. The echocardiography training has evolved over the years. In 2003, the first annual functional echocardiography course was held. Both neonatologists and cardiologists taught this 5-day course on ultrasound physics, imaging, and hemodynamics. Although our fellows were the primary target audience, the attendees included local, national, and international neonatologists and physicians in training. Since 2012, we have added echocardiography simulator training to enhance and accelerate the initial skill development.^14^ The fellows are required to attend the annual course in the first year of their fellowship. The post-course training has changed from an elective to mandatory since 2018. Each fellow is required to spend two weeks a year dedicated to learning echocardiography. This involves hands-on training on the simulator, performing echocardiography in the newborn nursery and NICU, and observation and case discussion in the echo lab. The simulation training prepares the fellow for the actual echocardiography and is especially important for the novice, although it is helpful for the more advanced learners.^15^ In addition, the trainees can improve their skills in differentiating normal from abnormal heart structure by imaging over 30 cases of various CHD cases on the simulator. Depending on the competency level, they will perform TNE throughout the year. A member of the TNE program with expertise in echocardiography or a pediatric cardiologist reviews each echocardiography study. By the end of the fellowship, each fellow has done over 100 complete echocardiograms. In addition, fellows participate in monthly hemodynamic case conferences and quality improvement meetings, where they learn the interpretation of echocardiography images and how this knowledge can be applied in clinical practice.

### Training after fellowship

This can be in the form of an additional year of fellowship dedicated to echocardiography and hemodynamics or a less structured and perhaps with a more limited scope for the practicing neonatologist. The first one-year hemodynamic fellowship program was established at the University of Iowa in 2019, with few other programs offering similar programs since. This approach had some advantages over training during the neonatology fellowship. The whole year is dedicated to learning TNE, understanding hemodynamics, and applying the knowledge in daily patient care. These programs typically have leading hemodynamic experts on the faculty and a robust supportive infrastructure. The graduates from these programs are expected to master TNE and continue developing hemodynamic expertise. However, this approach in training also has limitations. Typically, the programs take one fellow a year, and since there are only a handful of such programs, currently the impact of such training on spreading skills among neonatologists is limited. However, given the selection process for the most dedicated candidate and the comprehensive curriculum, the graduates from these programs are likely to have greater potential to initiate and establish a TNE program after graduation.

### Economic implications

Recruiting experts in TNE and providing them adequate time to introduce the concept to pediatric residents and provide dedicated training to fellows requires a financial commitment from the Departments of Pediatrics for both models. The additional year of advanced hemodynamics fellowship is currently not approved by ACGME and will require funding from Departments although these providers have the option of “moonlighting” and earn extra-service payments. Finally, an incentive (higher salary, guaranteed protected time, or earlier promotion to a higher rank) must be provided to graduating neonatal trainees with expertise in TNE.

### Policy change implications

With proposals to reduce time spent in the ICUs by ACGME,^16^ incoming fellows to neonatal-perinatal medicine are likely to need longer clinical time to achieve basic neonatal skills such as resuscitation, line placement, and intubation. The added burden of learning and developing expertise in TNE may limit the time available for scholarly activities in the “training during fellowship” model.

The recent suggestion by the National Academies of Science, Engineering, and Medicine (NASEM) to reduce the duration of pediatric fellowships to two years for clinically focused providers may lead to less time for additional TNE training during fellowships and support the “training after fellowship” model. However, the main focus of the NASEM report was pediatric subspecialties with difficulties in recruitment such as infectious diseases and endocrinology, and not neonatal perinatal medicine.^17^

Close attention should be paid to these proposals and their implementation as it is likely to impact the time available for TNE training.

## Conclusions

In the last decade, significant advances in hemodynamic evaluation, including the use of TNE, functional or point-of-care echocardiography, and cardiac POCUS, have been made in neonatology. The models of hemodynamic assessment using TNE by the primary neonatologist and neonatology consultation team are complementary and are needed to advance patient care. With increased recognition of the role of echocardiography in improving neonatal outcomes and increased demand for training, the important question arises of how neonatologists can be empowered with echocardiography skills while maintaining quality assurance. Evaluation of various models of developing this expertise by the national organizations tasked with pediatric residency and fellowship curricula might streamline the acquisition of TNE/POCUS skills to enhance care for critically ill neonates.

## Supplementary information

Supplementary Vedio 1

Supplementary Vedio 1 legend

## References

[1] Wu, T. W. & Noori, S. Recognition and management of neonatal hemodynamic compromise. *Pediatr. Neonatol.* **62**, S22–S29 (2021). 33485823 10.1016/j.pedneo.2020.12.007

[2] Rahde Bischoff, A. et al. Targeted neonatal echocardiography in patients with hemodynamic instability. *Pediatrics* **150**, e2022056415I (2022). 36317979 10.1542/peds.2022-056415I

[3] Kluckow, M., Seri, I. & Evans, N. Functional echocardiography: an emerging clinical tool for the neonatologist. *J. Pediatr.* **150**, 125–130 (2007). 17236886 10.1016/j.jpeds.2006.10.056

[4] Mertens, L. et al. Targeted Neonatal Echocardiography in the Neonatal Intensive Care Unit: practice guidelines and recommendations for training. Writing Group of the American Society of Echocardiography (ASE) in collaboration with the European Association of Echocardiography (EAE) and the Association for European Pediatric Cardiologists (AEPC). *J. Am. Soc. Echocardiogr. Publ. Am. Soc. Echocardiogr.* **24**, 1057–1078 (2011). 10.1016/j.echo.2011.07.014 21933743

[5] Boyd, S. & Kluckow, M. Point of care ultrasound in the neonatal unit: Applications, training and accreditation. *Early Hum. Dev.* **138**, 104847 (2019). 31488312 10.1016/j.earlhumdev.2019.104847

[6] Singh, Y. et al. Expert consensus statement ’Neonatologist-performed Echocardiography (NoPE)’-training and accreditation in UK. *Eur. J. Pediatr.* **175**, 281–287 (2016). 26362538 10.1007/s00431-015-2633-2

[7] de Boode, W. P. et al. Recommendations for neonatologist performed echocardiography in Europe: Consensus Statement endorsed by European Society for Paediatric Research (ESPR) and European Society for Neonatology (ESN). *Pediatr. Res.* **80**, 465–471 (2016). 27384404 10.1038/pr.2016.126 PMC5510288

[8] Singh, Y., Bhombal, S., Katheria, A., Tissot, C. & Fraga, M. V. The evolution of cardiac point of care ultrasound for the neonatologist. *Eur. J. Pediatr.* **180**, 3565–3575 (2021). 34125292 10.1007/s00431-021-04153-5

[9] Singh, Y. et al. International evidence-based guidelines on Point of Care Ultrasound (POCUS) for critically ill neonates and children issued by the POCUS Working Group of the European Society of Paediatric and Neonatal Intensive Care (ESPNIC). *Crit. Care Lond. Engl.* **24**, 65 (2020). 10.1186/s13054-020-2787-9 PMC7041196 32093763

[10] McNamara, P. J., Barker, P., Jain, A. & Lai, W. W. Towards use of POCUS to evaluate hemodynamics in critically ill neonates: caution before adoption in this population. *Crit. Care Lond. Engl.* **25**, 92 (2021). 10.1186/s13054-020-03394-4 PMC7927214 33658049

[11] Elsayed, Y. et al. Point-of-care ultrasound (POCUS) protocol for systematic assessment of the crashing neonate-expert consensus statement of the international crashing neonate working group. *Eur. J. Pediatr.* **182**, 53–66 (2023). 36239816 10.1007/s00431-022-04636-z PMC9829616

[12] Yousef, N., Singh, Y. & De Luca, D. Playing it SAFE in the NICU” SAFE-R: A targeted diagnostic ultrasound protocol for the suddenly decompensating infant in the NICU. *Eur. J. Pediatr.* **181**, 393–398 (2022). 34223967 10.1007/s00431-021-04186-w PMC8256195

[13] Nguyen, J., Amirnovin, R., Ramanathan, R. & Noori, S. The state of point-of-care ultrasonography use and training in neonatal-perinatal medicine and pediatric critical care medicine fellowship programs. *J. Perinatol. J. Calif. Perinat. Assoc.* **36**, 972–976 (2016). 10.1038/jp.2016.126 27513327

[14] Siassi, B. et al. Virtual Neonatal Echocardiographic Training System (VNETS): An echocardiographic simulator for training basic transthoracic echocardiography skills in neonates and infants. *IEEE J. Transl. Eng. Health Med.* **6**, 4700113 (2018). 30464863 10.1109/JTEHM.2018.2878724 PMC6242698

[15] Noori S. et al. Effectiveness of simulation training in acquiring echocardiography skills among neonatology care providers. *Am. J. Perinatol*. Published online June 6, 2022. 10.1055/a-1845-2083 10.1055/a-1845-2083 35523411

[16] ACGME Program Requirements for Graduate Medical Education in Pediatrics. Review and Comment (ACGME.Org) 320_Pediatrics_Rc_022023 (Acgme.Org).

[17] The Future Pediatric Subspecialty Physician Workforce: Meeting the Needs of Infants, Children, and Adolescents. National Academies of Sciences, Engineering, and Medicine. 2023. (The National Academies Press., 2023). 10.17226/27207. 38295208

