# Supplementary material for: Hemodynamic assessment by neonatologist using echocardiography: Primary provider versus consultation model
Source: Pediatr Res. 2024 May 22;96(7):1603–8. doi: 10.1038/s41390-024-03248-7 (PMC11772219; doi:10.1038/s41390-024-03248-7)
Supplement: Supplementary file 2 — Supplementary Vedio 1 legend [file 41390_2024_3248_MOESM2_ESM.docx]

**Video 1.** A two-week-old preterm infant with severe necrotizing enterocolitis had no measurable blood pressure after exploratory laparotomy despite receiving dopamine 15 mcg/kg/min and multiple normal saline boluses. A point-of-care echocardiography showed poor myocardial contractility. After starting dobutamine and administering an intravenous calcium bolus for low calcium level, blood pressure and contractility improved.
